# Pharmacokinetics of Acetaminophen and Metformin Hydrochloride in Rats After Exposure to Simulated High Altitude Hypoxia

**DOI:** 10.3389/fphar.2021.692349

**Published:** 2021-06-18

**Authors:** Jun-bo Zhu, Jian-xin Yang, Yong-qiong Nian, Gui-qin Liu, Ya-bin Duan, Xue Bai, Qian Wang, Yang Zhou, Xue-jun Wang, Ning Qu, Xiang-yang Li

**Affiliations:** ^1^ Research Center for High Altitude Medicine, Qinghai University Medical College, Xining, China; ^2^ State Key Laboratory of Plateau Ecology and Agriculture, Qinghai University, Xining, China; ^3^ School of Pharmacy, Qinghai Nationalities University, Xining, China; ^4^ College of Eco-Environmental Engineering, Qinghai University, Xining, China; ^5^ Department of Anesthesiology, Red Cross Hospital of Qinghai, Xining, China; ^6^ Department of Anesthesiology, Qinghai Hospital of Traditional Chinese Medicine, Xining, China

**Keywords:** acetaminophen, metformin hydrochloride, high altitude hypoxia, pharmacokinetics, UGT1A1, OCT2

## Abstract

The pharmacokinetic characteristics of drugs were altered under high altitude hypoxia, thereby affecting the absorption, distribution, metabolism, and excretion of drug. However, there are few literatures on the pharmacokinetic changes of antipyretic and pain-relieving drugs and cardiovascular system drugs at high altitude. This study aimed to evaluate the pharmacokinetics of acetaminophen and metformin hydrochloride in rats under simulated high altitude hypoxia condition. Mechanically, the protein and mRNA expression of uridine diphosphate glucuronyltransferase 1A1 (UGT1A1) and organic cation transporter 2 (OCT2) were investigated by enzyme linked immunosorbent assay (ELISA) and quantitative real-time polymerase chain reaction (qRT-PCR), respectively. Compared with the normoxia group, the t_1/2_ and AUC of acetaminophen were significantly increased, and the CL/F was significantly decreased in rats after exposure to simulated high altitude hypoxia. The t_1/2_ of metformin hydrochloride was significantly increased by simulated high altitude hypoxia. No significant differences in AUC and CL/F of metformin hydrochloride were observed when comparing the hypoxia group with the normoxia group. The protein and mRNA expression of UGT1A1 and OCT2 were decreased significantly under hypoxia in rats. This study found obvious changes in the pharmacokinetics of acetaminophen and metformin hydrochloride in rats after exposure to simulated high altitude hypoxia, and they might be due to significant decreases in the expressions of UGT1A1 and OCT2. To sum up, our data suggested that the pharmacokinetics of acetaminophen and metformin hydrochloride should be reexamined, and the optimal dose should be reassessed under hypoxia exposure.

## Introduction

The environmental characteristics of high altitude is featured by hypoxia, low pressure, low temperature, dry air, strong wind, and strong ultraviolet radiation, in which hypoxia plays a major role in affecting the activity of human life (Li J. et al., 2015). High altitude hypoxia influences the living quality of 140 million people at high altitude and affects numerous people who travel to high altitudes for tourism, military and sport, among other reasons (Jiang et al., 2014). High altitude hypoxia exerts different effects on the digestive system, cardiovascular and cerebrovascular system, nervous system, and exogenous substance metabolism (Aronwisnewsky et al., 2012; Kimura and Sadek, 2012; Adak et al., 2014; Whayne, 2014), which in turn cause high altitude sickness including acute mountain sickness, chronic polycythemia, cardivascular disease, pulmonary edema, and cerebral edema (Ronen et al., 2014; Mishra et al., 2015; Riley and Gavin, 2017).

High altitude hypoxia-related research mainly focus on the impact of the hypoxia environment on the body and the prevention and treatment of high altitude diseases (Li et al., 2011; Zhang and Li, 2015). In recent years, the changes of drug pharmacokinetics in high altitude areas have attracted wide attention of scholars (Zhou et al., 2018). The physiologic changes caused by high altitude hypoxia may alter the absorption, distribution, metabolism, and excretion of drugs, which may require adjustment in dosage regimens to prevent toxicity or maintain efficacy in the high altitude hypoxia environment (Zhang and Li, 2015; Zhou et al., 2018). Studies have demonstrated the pharmacokinetic differences of drugs such as Lidocaine hydrochloride (Zhang et al., 2016), sulfamethoxazole (Li et al., 2011; Li et al., 2009), lithium (Arancibia et al., 2003), propranolol (Li J. et al., 2015), metoprolol (Zhang et al., 2014), and ibuprofen (Gola et al., 2013; Gola et al., 2016) at high altitudes. These studies indicated that the metabolism of most drugs under the high altitude hypoxia environment slowed down, and mean residence time (MRT), half-life time (t_1/2_) and area under the drug-time curve (AUC) was increased, and clearance rate (CL) was reduced. At present, the research on the effects of hypoxia on drug metabolism is mainly concentrated on the prevention and treatment of high altitude disease drugs, while few literatures on the pharmacokinetic changes of antipyretic and pain-relieving drugs and cardiovascular system drugs under hypoxic conditions was reported.

Acetaminophen is the most widely used acetanilide antipyretic analgesic currently, and its overuse and abuse can cause serious adverse reactions and hepatotoxicity (Bhushan et al., 2014). The blood concentration of acetaminophen are monitored as one of the routine tests for emergency poisoning patients (Mattia and Coluzzi, 2009). Acetaminophen is metabolized mainly by the uridine diphosphate glucuronyltransferase 1A1 (UGT1A1) in the liver (Wang et al., 2011). Metformin hydrochloride, one of the most commonly used drugs in clinic for the treatment of type 2 diabetes, mainly absorbed from the small intestine and not metabolized by the liver (Klip and Leiter, 1990). The metabolism of metformin hydrochloride are mainly affected by organic cation transporter 2 (OCT2) (Kimura et al., 2005). The pharmacokinetics of acetaminophen and metformin hydrochloride has not reported in the high altitude hypoxia environment. For these reasons, we determined the pharmacokinetics of acetaminophen and metformin hydrochloride under high altitude hypoxia in rats, and the protein and mRNA expression of UGT1A1 and OCT2 were evaluated, respectively, in order to give information about rational drug use at high altitude.

## Materials and Methods

### Reagents and Instruments

Acetaminophen reference substance was obtained from Tokyo Chemical Industry Co., Ltd. (Lot: 5IXZK-HA, Tokyo, Japan). Acetaminophen bulk drug was purchased from Henan Xiaoyang Biotechnology Co., Ltd. (Lot: 011802115, Zhengzhou, China). The reference substance of metformin hydrochloride and 1-Dodecanesulfonic acid sodium salt was obtained from J and K Scientific Co., Ltd. (Lot: L290Q101, LE80P54, Beijing, China). The raw material of metformin hydrochloride was obtained from Hubei Henglvyuan Technology Co., Ltd. (Lot: 10180133, Changsha, China). HPLC-grade methanol and acetonitrile were both obtained from Shandong Yuwang Company, Inc. (Jinan, China). All chemicals and solvents were at the highest grade of purity available. Rat UGT1A1 and OCT2 ELISA kits were purchased from Dongfang Tuojin Technology Co., Ltd. (Lot: 20180501, 20180628, Beijing, China). RNAiso Plus and PrimeScript™ RT Reagent were purchased from Takara (Kyoto, Japan). The primers used in qRT-PCR were synthesized by Takara. SYBR^®^ Premix Ex Taq™ Kit was purchased from Takara (Kyoto, Japan).

### Animals and Experimental Treatments

Male Sprague Dawley rats weighing 180–220 g were obtained from the laboratory animal center of Xi’an Jiaotong University Medical College (certificate No.: 2007-001, Xi’an, China). All rats were housed per cage in separate rooms and given ad libitum access to water and food pellets, and were adapted for a week with a constant temperature (23 ± 2°C) and humidity (55 ± 5%) under a cycle of 12 h of light and dark. All experimental procedures were applied in strict accordance with the National Institutes of Health Guide for the Care and Use of Laboratory Animals.

Twenty-four rats were randomly divided into normoxia group (390 m, PaO_2_: 20.2 kPa), acute hypoxia group with simulated high altitude (5,000 m, PaO_2_: 11.1 kPa) and chronic hypoxia group with simulated high altitude (5,000 m, PaO_2_: 11.1 kPa), and every group had eight male rats. Rats in the normoxia group lived at an altitude of ≈390 m in Xi’an, Shanxi Province, northwest of China. Rats in the acute hypoxia group and the chronic hypoxia group received a 72-h acute hypoxia exposure and a 30-day chronic hypoxia exposure at simulated an altitude of 5,000 m and an oxygen partial pressure of 11.1 kPa inside a hypobaric chamber (DXY300, Guizhou Fenglei Aviation Ordnance Co., Ltd., Anshun, China).

### Determination of Physiologic and Blood Parameters

To verify the responses of rats after exposure to high altitudes, capillary oxygen saturation (ScO_2_) was measured using a TuffSat TTM Oximeter (GE-Datex Ohmeda, United States). A routine blood examination contains red blood cells, white blood cells, hemoglobin, lymphocyte levels, granulocyte, and platelet count were determined with an XFA6100 automatic hematology analyzer (Nanjing Perlove Medical Equipment, Co., Ltd., China). Blood biochemical parameters comprises ScO_2_, alanine aminotransferase, aspartate aminotransferase, total bilirubin, total protein, albumin, glutamate, total cholesterol, and globulin were examined by an AU2700 automatic biochemistry analyzer (Olympus Corporation, Japan) in rats after exposure to simulated high altitude hypoxia.

### Study Design of Pharmacokinetics

To evaluate the effect of high altitude hypoxia on pharmacokinetics of acetaminophen, rats from the normoxia group, acute and chronic hypoxia groups were orally administrated with acetaminophen solution at a dose of 105 mg/kg after an overnight fast of ≥12 h (with water allowed ad libitum). 0.3 ml of serial blood samples were collected from the eye socket before (baseline) and 10, 30 min, 1, 1.5, 2, 3, 4, 6, 8, and 12 h after the study drug administration. To evaluate the effect of high altitude hypoxia on pharmacokinetics of metformin hydrochloride, rats from the normoxia group, acute and chronic hypoxia groups were orally administrated with metformin hydrochloride solution at a dose of 45 mg/kg after an overnight fast of ≥12 h (with water allowed ad libitum). 0.3 ml of serial blood samples were collected from the eye socket before (baseline) and 10, 30 min, 1, 1.5, 2, 3, 4, 6, 8, and 12 h after the study drug administration. All blood samples were centrifuged at 3,000 rpm for 15 min at 4°C and the plasma were separated and then immediately stored at −20°C.

### Bioanalytical Method

The concentration of acetaminophen and metformin hydrochloride in plasma were assayed by HPLC. 120 μl of the plasma sample was placed in a 0.5 ml centrifuge tube, and added 40 μl of 30% perchloric acid. Then, vortexed the sample for 1 min and centrifuged at 16,000 rpm for 10 min at 4°C, and injected 20 μL of the supernatant into the HPLC system to record the peak area of the chromatogram.

HPLC analyses of acetaminophen and metformin hydrochloride were performed with a HPLC pump (1,260 Infinity II, Agilent Technologies Co., Ltd., California, United States) and a diode array detection system (1,260 Infinity II, Agilent Technologies Co., Ltd., California, United States) equipped with a C18 column (Boston Green, Boston Analytics, Inc., Boston, United States; 4.6 × 250 mm, inner diameter, 10 μm) with a constant column temperature at 25°C. The mobile phase of acetaminophen was methyl alcohol-water (30∶70, v/v) with a constant flow rate of 1.0 ml/min. The detection wavelength was at 248 nm. The linearity range of the method is 0.5–40 μg/ml, and the correlation coefficients of the regression lines were always >0.9990. The lower limit of quantitation was 0.5 μg/ml. The intra-day CV of spiked quality-control samples was 8.51, 3.88, 0.98, and 1.37%, respectively, and the inter-day CV of spiked quality-control samples was 8.16, 3.92, 0.92, and 1.16% for acetaminophen concentrations of 0.5, 1, 18, and 36 μg/ml, respectively. The accuracy were respectively measured as −6.00, 3.00, −3.28, and 1.08% for the intra-day accuracy and −2.00, 2.00, −3.06 and −0.08% for the inter-day accuracy at the different target concentrations.

The mobile phase of metformin hydrochloride was 3 mmol/l sodium dodecyl sulfate-acetonitrile-triethylamine (68∶32∶0.5, v/v/v, pH = 2.0 with phosphoric acid) with a constant flow rate of 1.0 ml/min. The detection wavelength was at 254 nm. The linearity range of the method is 0.5–10 μg/ml, and the correlation coefficients of the regression lines were always >0.9990. The lower limit of quantitation was 0.5 μg/ml. The intra-day CV of spiked quality-control samples was 4.55, 1.37, 2.86 and 0.69%, respectively, and the inter-day CV of spiked quality-control samples was 4.44%, 2.74%, 2.84%, and 1.16% for acetaminophen concentrations of 0.5, 0.8, 3, and 8 μg/ml, respectively. The accuracy were respectively measured as −12.00, −8.75, 6.67, and −3.22% for the intra-day accuracy and −10.00, −8.75, −6.00, and 3.89% for the inter-day accuracy at the different target concentrations.

### Pharmacokinetic Analysis

Pharmacokinetic values for acetaminophen and metformin hydrochloride were calculated for each rat, from which mean values were then determined for analysis. The elimination rate constant (Ke), half-life time (t_1/2_), mean residence time (MRT), volume of distribution (Vd/F), clearance (CL/F), and area under the plasma concentration-time curve (AUC) were calculated under a noncompartmental analysis using DAS 2.0 software (Institute of Clinical Pharmacology, Shanghai University of Traditional Chinese Medicine, Shanghai, China). The values of peak time (T_max_) and peak concentration (C_max_) were obtained from the original data directly.

### Preparation of Rat Liver Microsomes and Renal Tissue

To evaluate the regulation of simulated high altitude hypoxia on the protein expression levels of UGT1A1, liver tissues of each rat were collected after collecting the last blood sample. Liver microsomes were prepared by differential centrifugation, as previously reported (Zhang and Li, 2015; Zhang and Li, 2015; Zhou et al., 2018). Briefly, an appropriate amount of liver samples were weighed, and two volumes of 50 mmol/l ice-cold Tris-Cl buffer (pH 7.4, 0.25 M sucrose) were then added. The minced tissue was homogenized with an automatic homogenizer at 500 rpm and centrifuged at 10,000 rpm for 30 min at 4°C using a high speed centrifuge. The supernatant was then collected and centrifuged at 100,000 rpm for 80 min at 4°C using an ultracentrifuge (Optima MAX-XP, Beckman Coulter Inc., California, United States). The supernatant was discarded, and the microsome pellet was resuspended in a homogenization medium. The 0.5 ml of liver microsome suspension was aliquoted into Eppendorf tubes and stored at -80°C until use.

To evaluate the regulation of simulated high altitude hypoxia on the protein expression levels of OCT2, renal tissues of each rat were collected after collecting the last blood sample. An appropriate amount of renal samples were weighed, and two volumes of 50 mmol/l ice-cold Tris-HCl buffer (pH 7.4, 0.25 M sucrose) were then added. The tissue was homogenized with an automatic homogenizer at 500 rpm and centrifuged at 2,000 rpm for 30 min at 4°C using a high speed centrifuge (TGL-16B, Anting Scientific Instrument Factory, Shanghai, China). The 0.5°ml of renal tissues suspension was aliquoted into Eppendorf tubes and stored at −80°C until use.

### ELISA Analysis of UGT1A1 and OCT2 Proteins

Diluted the standard according to the kit specification, the 50 μl of standard and the 40 μl of sample dilution solution were added to the standards wells and sample wells, respectively, then 10 μl of the sample was added to the sample wells. There were no sample and enzyme reagent in the blank hole. It was then incubated at 37°C for 30 min. After discarding the liquid, each well was filled with washing buffer, allowed to stand for 30 s, and discarded, repeated for a total of five washes. Then, 50 μl of the enzyme labeling reagent was added to each well, and mixing, it was incubated at 37°C for 30 min. After the washing was completed, 50 μl of the developer A and 50 μl of the developer B were added to each well, and the mixture was allowed to react for 15 min away from light. The reaction was terminated by the addition of 50 μl of stop solution, and the absorbance OD value was measured at a wavelength of 450 nm using a Spectrophotometer. The concentration of UGT1A1 and OCT2 in the sample was then calculated by a standard curve. The equation of the standard curve for UGT1A1 was C = 55.922 × O.D. −3.4473. The correlation coefficients of regression line were >0.9990 in the range of 1–100 ng/ml. The lower limit of quantitation was 1 ng/ml. The CV of intraday precision was less than 10%, and the CV of interday precision was less than 12% for low, middle, and high-level UGT1A1. The equation of the standard curve for OCT2 was C = 16.826 × O.D. −1.8297. The correlation coefficients of regression line were >0.9960 in the range of 1.2–24 ng/ml. The lower limit of quantitation was 1.2 ng/ml. The CV of intraday precision was less than 10%, and the CV of interday precision was less than 12% for low, middle, and high-level OCT2.

### RNA Isolation and Real-Time PCR Analysis of UGT1A1 and OCT2 mRNA

The liver and kidney of eight rats from every group were excised immediately after death and stored at −80°C before using. Approximately 100–200 mg of liver and renal tissue was homogenized and total RNA was extracted by Trizol reagent. The concentration and purity of RNA solution was determined using an ultraviolet spectrophotometer. The primers and reaction protocol for rat UGT1A1 and OCT2 were designed by Major Bio-pharm Technology Co., Ltd (Shanghai, China). All real-time PCRs were carried out using a SYBR^®^ Premix Ex Taq™ Kit (Takara) in accordance with the manufacturer’s instructions. Amplification was performed in PCR capillaries on a CFX Connect real-time detection system (Bio-Rad, United States). The PCR program settings are as follows: pre-denatured cycle (1 cycle): 95°C for 5 min, PCR cycle (40 cycles): 95°C for 10 s, 60°C for 30 s, and dissolution curve: 60–95°C for 30 s, and increase the set point temperature after cycle two by 0.5°C. Fold induction values were calculated using the value of 2^−ΔΔCt^.

UGT1A1 primer sequence:

5′-AGG​AGA​GCT​ACC​ATG​TCC​GT-3′ (forward primer)

5′-CTC​CGA​GCA​TAC​TCA​GCC​AG-3′ (reverse primer)

OCT2 primer sequence:

5′-TGA​GGA​CGC​TGG​CAA​GAA​AT-3′ (forward primer)

5′-TGA​GGA​CGC​TGG​CAA​GAA​AT-3′ (reverse primer)

*β*-actin primer sequence:

5′-GCT​ATG​TTG​CCC​TAG​ACT​TCG​A-3′ (forward primer)

5′-GAT​GCC​ACA​GGA​TTC​CAT​ACC-3′ (reverse primer)

#### Statistical Analysis

Data are expressed as the mean ± SD values. Statistical data were processed using One-way analysis of variance (ANOVA) and SPSS 23.0 software, and the least significant difference test was used for two-group comparison. Values of *p* < 0.05 were considered statistically significant.

## Results

### HPLC Method Validation

The retention times for acetaminophen and metformin hydrochloride were respectively measured as approximately 7.0 and 10.0 min under the present analytical conditions. The peaks of acetaminophen and metformin hydrochloride were well-differentiated, and no endogenous interference in the rat plasma was observed. Strong levels of acetaminophen linearity were achieved within a range of 0.5–40 μg/ml with all coefficients of correlation exceeding 0.9990. Intraday precision and accuracy levels ranged from 0.98 to 8.51% and −6.00 to 3.00%, respectively. Interday precision and accuracy levels in the rat plasma ranged from 0.92 to 8.16% and from −3.06 to 2.00%, respectively. Strong levels of metformin hydrochloride linearity were achieved within a range of 0.5–10 μg/ml with all coefficients of correlation exceeding 0.9990. Intraday precision and accuracy levels ranged from 0.69 to 4.55% and from −12.00 to 6.67%, respectively. Interday precision and accuracy levels in the rat plasma ranged from 1.16 to 4.44% and from −10.00 to 3.89%, respectively.

### Physiologic and Blood Parameters

**FIGURE 1:**
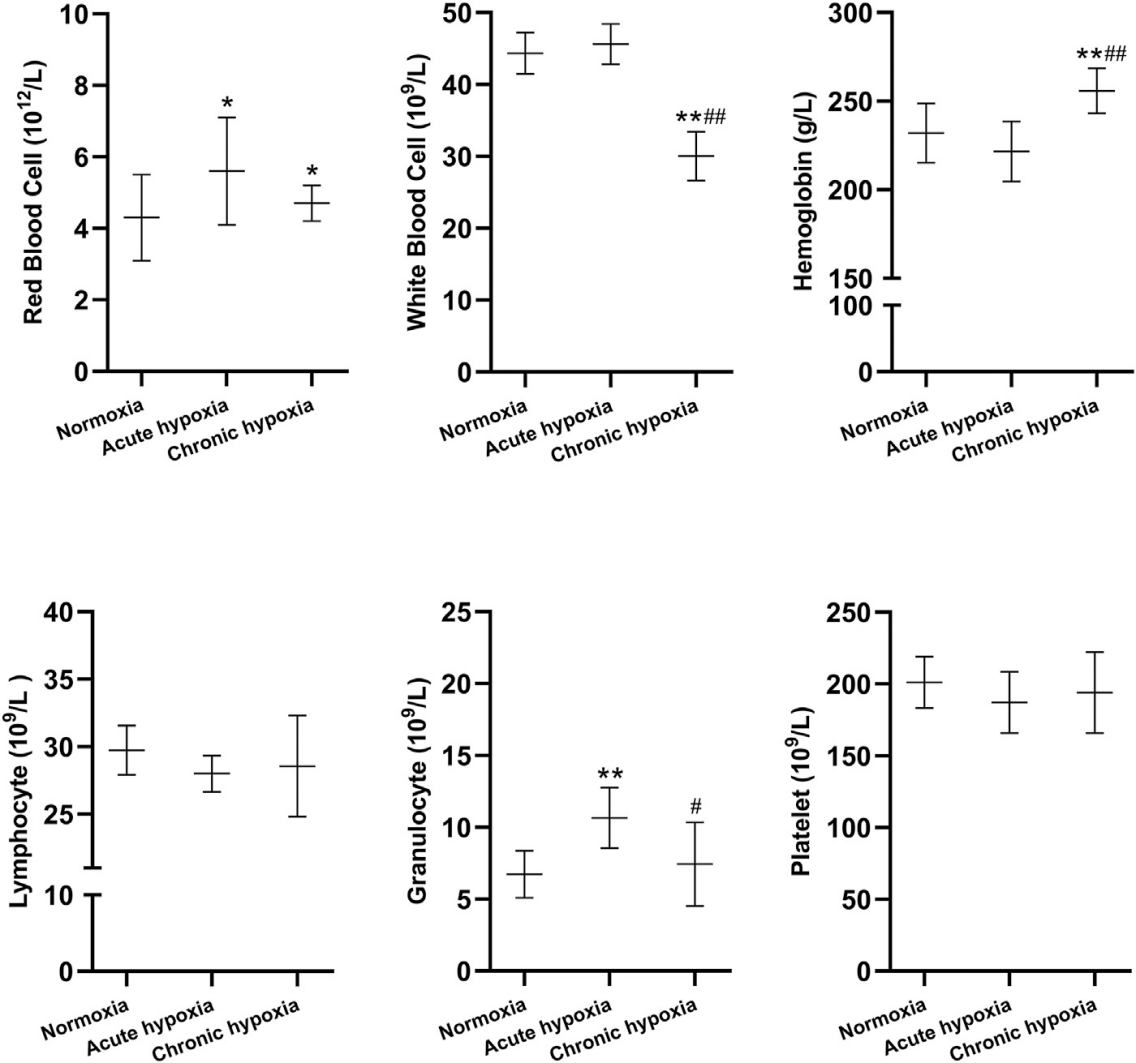
Changes in red blood cells, white blood cells, hemoglobin, lymphocytes, granulocytes, and platelets in the rats after exposure to simulated high altitude hypoxia. The data are presented as mean ± SD. *N* = 8. The data were analyzed using ANOVA, and the differences between the means of two groups were compared using LSD tests. ^*^ *p* < 0.05, **p < 0.01 compared to the normoxia group; ^#^ *p* < 0.05, ^##^ *p* < 0.01 compared to the acute hypoxia group.

**FIGURE 2:**
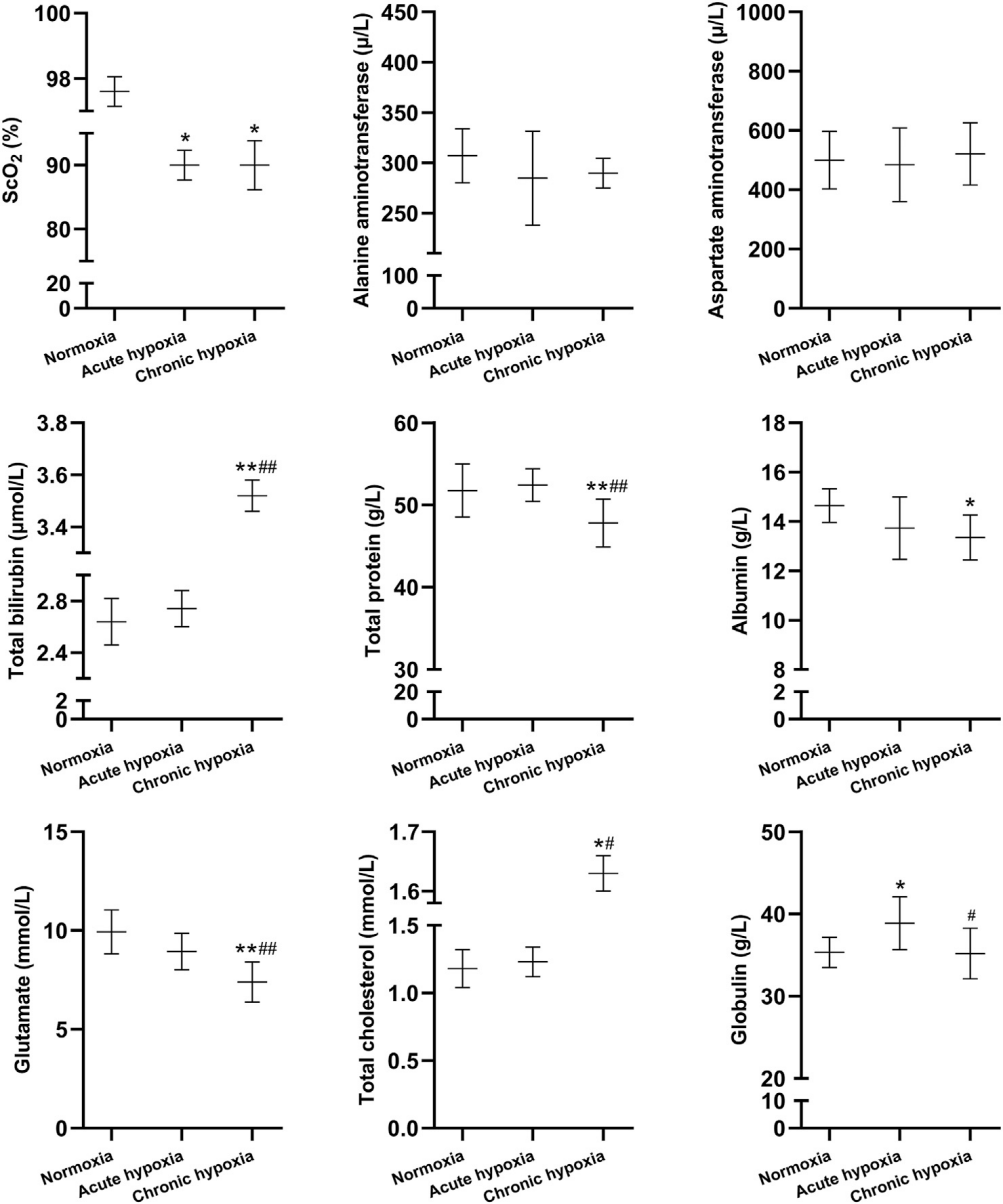
Changes in ScO2, alanine aminotransferase, aspartate aminotransferase, total bilirubin, total protein, albumin, glutamate, total cholesterol, and globulin in the rats after exposure to simulated high altitude hypoxia. The data are presented as mean ± SD. *N* = 8. The data were analyzed using ANOVA, and the differences between the means of two groups were compared using LSD tests. ^*^ *p* < 0.05, **p < 0.01 compared to the normoxia group; ^#^ *p* < 0.05, ^##^ *p* < 0.01 compared to the acute hypoxia group.

Changes in blood routine parameters and biochemical blood tests of rats induced by simulated high altitude hypoxia are shown in Figures 1, 2. The red blood cell count were 34.9 and 14.0% higher in the acute and chronic hypoxia groups compared to the normoxia group (both *p* < 0.05), respectively. The white blood cell count was 32.2% lower in the chronic hypoxia group than in the normoxia group (*p* < 0.01), and 34.0% lower in the chronic hypoxia group than in the acute hypoxia group (*p* < 0.01). The hemoglobin was 10.3% higher in the chronic hypoxia group than in the normoxia group (*p* < 0.01), and 19.4% lower in the chronic hypoxia group than in the acute hypoxia group (*p* < 0.01). Compared to the normoxia group, the granulocyte levels were 59.7% higher in the acute hypoxia groups (*p* < 0.01), respectively, and 30.8% lower in the chronic hypoxia group than in the acute hypoxia group (*p* < 0.05). For lymphocyte levels and platelet count, there was no significant difference when comparing the acute or chronic hypoxia groups with the normoxia group.

Compared to the normoxia group, the S_C_O_2_ value was decreased by 7.8 and 7.8% in the acute and chronic hypoxia group (both *p* < 0.05), respectively. Total bilirubin was increased by 33.3% in the chronic hypoxia group than in the normoxia group (*p* < 0.01), and increased by 28.5% in the chronic hypoxia group than in the acute hypoxia group (*p* < 0.01). Total protein was decreased by 7.6% in the chronic hypoxia group than in the normoxia group (*p* < 0.01), and decreased by 8.8% in the chronic hypoxia group than in the acute hypoxia group (*p* < 0.01). Albumin was decreased by 8.8% in the chronic hypoxia group than in the normoxia group (*p* < 0.05). Compared to the normoxia group, the glutamate levels was decreased by 25.6% in the acute hypoxia groups (*p* < 0.01), and decreased by 17.3% in the chronic hypoxia group than in the acute hypoxia group (*p* < 0.01). Total cholesterol was increased by 25.0% in the chronic hypoxia group than in the normoxia group (*p* < 0.05), and increased by 24.3% in the chronic hypoxia group than in the acute hypoxia group (*p* < 0.05). Compared to the normoxia group, the globulin levels was increased by 10.0% in the acute hypoxia groups (*p* < 0.05), and decreased by 9.5% in the chronic hypoxia group than in the acute hypoxia group (*p* < 0.05). For alanine aminotransferase and aspartate aminotransferase, there was no significant difference when comparing the acute or chronic hypoxia groups with the normoxia group.

### Pharmacokinetic

**FIGURE 3:**
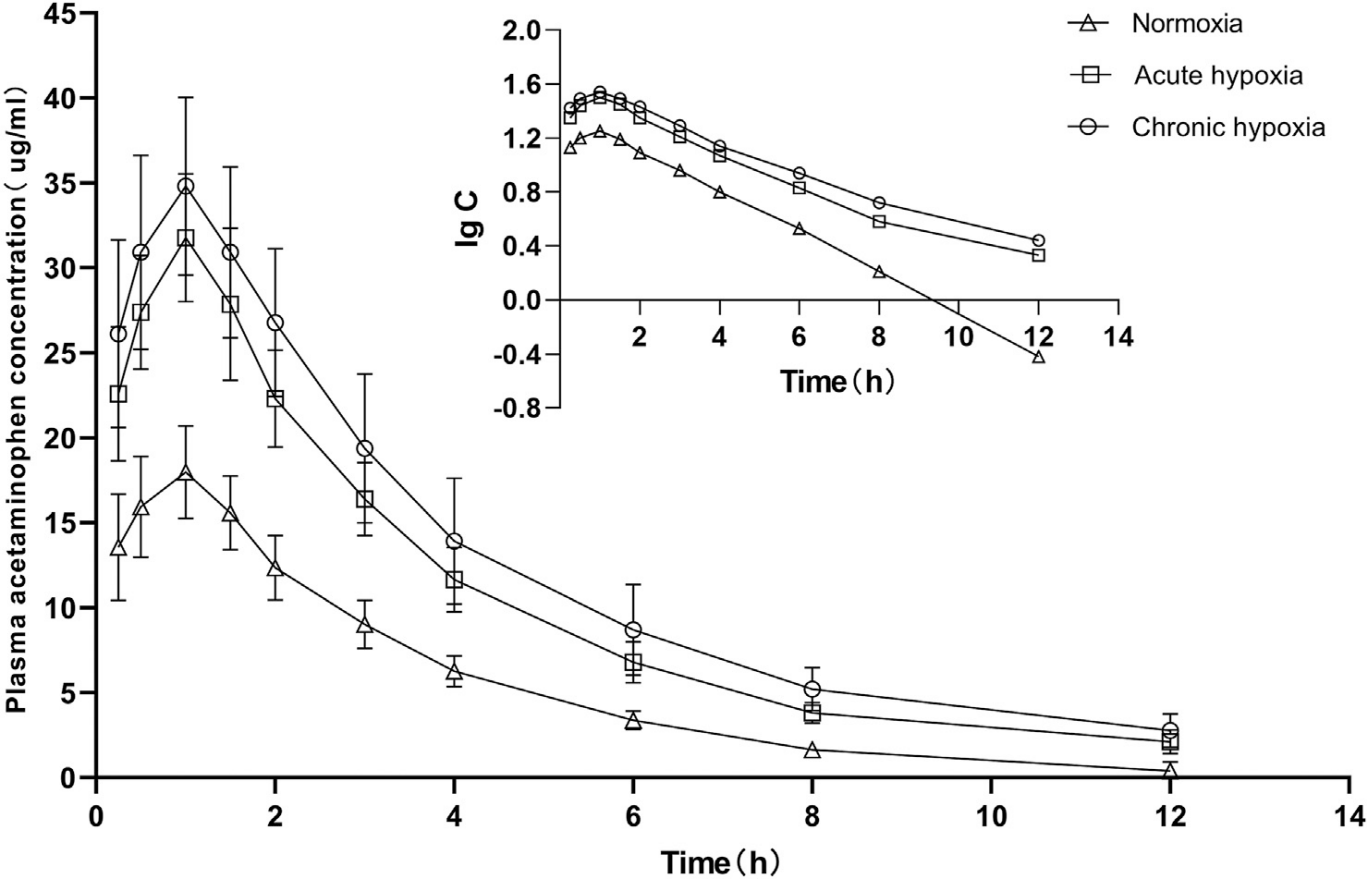
Mean plasma concentration time curve for oral acetaminophen (105 mg/kg) for rats after exposure to simulated high altitude hypoxia (*N* = 8).

**FIGURE 4:**
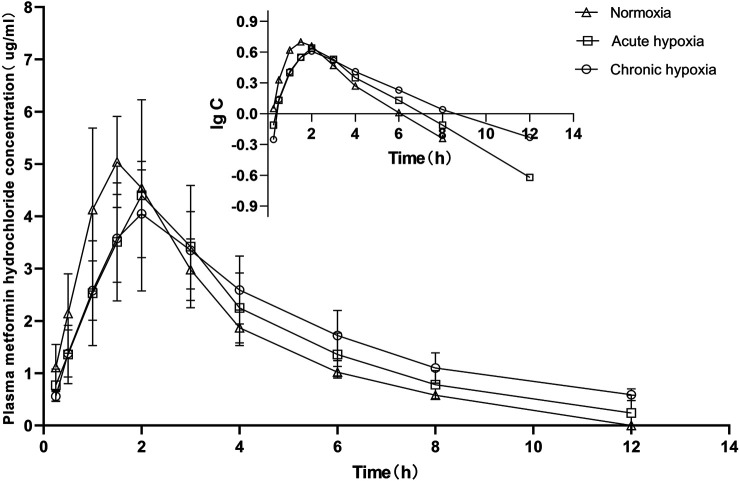
Mean plasma concentration time curve for oral metformin hydrochloride (45 mg/kg) for rats after exposure to simulated high altitude hypoxia (*N* = 8).

The pharmacokinetic parameters of acetaminophen and metformin hydrochloride were not significantly different between genders, and both data were combined. The mean pharmacokinetic parameters of acetaminophen and metformin hydrochloride of rat are shown in Tables 1, 2, respectively. Figures 3, 4 show mean plasma concentration time profiles for acetaminophen and metformin hydrochloride, respectively. Concentration time profiles for acetaminophen and metformin hydrochloride in plasma obtained for the three groups are similar in shape. Comparisons drawn between t_1/2_ in acetaminophen obtained from the rat plasma for the three groups studied revealed increases of 21.7 and 40.9% (both *p* < 0.01) after exposure to acute and chronic high altitude hypoxia, respectively, and an increase of 15.8% in the chronic hypoxia group than in the acute hypoxia group (*p* < 0.01). Compared to the normoxia group, MRT_0-12_ values were 19.6 and 24.6% higher in the acute and chronic hypoxia groups (both *p* < 0.01), respectively. We also observed increases of 1.9 and 2.3-fold for AUC_0-12_ in the acute and chronic hypoxia groups (both *p* < 0.01), respectively, compared to the normoxia group. CL/F values were 1.9 and 2.3-fold lower for the acute and chronic hypoxia groups (both *p* < 0.01), respectively, than for the normoxia group. The Vd/F value was 37.6 and 38.6% (*p* < 0.01) lower in the acute and chronic hypoxia groups, respectively, compared with the normoxia group. The Cmax value was 76.8 and 93.7% (*p* < 0.01) higher in the acute and chronic hypoxia groups, respectively, than in the normoxia group. For Tmax, there was no significant difference when comparing the acute or chronic hypoxia groups with the normoxia group.

**TABLE 1:** Pharmacokinetic parameters of acetaminophen in rat plasma after exposure to simulated high altitude hypoxia.

Parameters	Normoxia	Acute hypoxia	Chronic hypoxia
K_e_, 1/h	0.34 ± 0.02	0.29 ± 0.01^a^	0.24 ± 0.01^a^ ^,^ ^b^
t_1/2_, h	2.03 ± 0.11	2.47 ± 0.08^a^	2.86 ± 0.14^a^ ^,^ ^b^
MRT_(0–12)_, h	2.80 ± 0.31	3.35 ± 0.08^a^	3.49 ± 0.25^a^
CL/F, L/kg/h	1.60 ± 0.23	0.83 ± 0.11^a^	0.69 ± 0.14^a^
Vd/F, L/kg	4.68 ± 0.64	2.92 ± 0.41^a^	2.87 ± 0.64^a^
AUC_(0–12)_, h·μg/ml	64.22 ± 9.63	125.52 ± 17.75^a^	149.86 ± 29.95^a^
AUC_(0-∞)_, h·μg/ml	67.63 ± 10.20	129.75 ± 18.36^a^	158.22 ± 31.89^a^
AUMC_(0–12)_, h^2^·μg/ml	180.39 ± 35.15	419.48 ± 69.26^a^	525.59 ± 127.72^a^
AUMC_(0-∞)_, h^2^·μg/ml	230.28 ± 40.37	534.41 ± 105.60^a^	708.72 ± 184.89^a^
T_max_, h	1.02 ± 0.07	1.09 ± 0.09	1.08 ± 0.12
C_max_, μg/ml	17.97 ± 2.73	31.77 ± 3.76^a^	34.80 ± 5.24^a^

Data are expressed as the mean ± SD values (*n* = 8).

a *p* < 0.01 compared to the normoxia group;

b *p* < 0.01 compared to the acute hypoxia group. ke, elimination rate constant; t_1/2_, half-life; CL/F, total plasma clearance; Vd/F, apparent volume of distribution; Cmax, the maximum plasma concentration; AUC, area under the concentration time curve; AUMC, AUC of the first moment; MRT, mean residence time.

**TABLE 2:** Pharmacokinetic parameters of metformin hydrochloride in rat plasma after exposure to simulated high altitude hypoxia.

Parameters	Normoxia	Acute hypoxia	Chronic hypoxia
K_e_, 1/h	0.35 ± 0.03	0.27 ± 0.02^b^	0.22 ± 0.01^b^ ^,^ ^c^
t_1/2_, h	1.98 ± 0.14	2.53 ± 0.14^b^	3.10 ± 0.11^b^ ^,^ ^c^
MRT_(0–12)_, h	2.89 ± 0.14	3.60 ± 0.51^a^	4.44 ± 0.17^b^ ^,^ ^c^
CL/F, L/kg/h	2.47 ± 0.32	2.43 ± 0.66	2.01 ± 0.40
Vd/F, L/kg	7.08 ± 1.29	8.85 ± 2.31	8.97 ± 1.81
AUC_(0–12)_, h·μg/ml	17.40 ± 2.14	18.49 ± 6.62	21.85 ± 4.77
AUC_(0-∞)_, h·μg/ml	18.85 ± 2.18	20.38 ± 6.28	23.87 ± 5.30
AUMC_(0–12)_, h^2^·μg/ml	50.16 ± 5.44	69.16 ± 34.14	97.09 ± 22.08^b^
AUMC_(0-∞)_, h^2^·μg/ml	67.92 ± 6.24	99.16 ± 42.25	140.73 ± 28.69^b^
T_max_, h	1.62 ± 0.35	1.94 ± 0.56	2.01 ± 0.04
C_max_, μg/ml	5.44 ± 1.09	4.72 ± 1.58	4.05 ± 0.84^a^

Data are expressed as the mean ± SD values (*n* = 8).

a *p* < 0.05,

b *p* < 0.01 compared to the normoxia group.

c *p* < 0.01 compared to the acute hypoxia group. ke, elimination rate constant; t_1/2_, half-life; CL/F, total plasma clearance; Vd/F, apparent volume of distribution; Cmax, the maximum plasma concentration; AUC, area under the concentration time curve; AUMC, AUC of the first moment; MRT, mean residence time.

Relative to the value in the normoxia group, t_1/2_ values for metformin hydrochloride were 27.8 and 56.6% higher in the acute and chronic hypoxia groups (both *p* < 0.01), respectively, and 22.5% higher in the chronic hypoxia group than in the acute hypoxia group (*p* < 0.01). Compared to the normoxia group, MRT_0-12_ values were 24.6% (*p* < 0.05) and 53.6.6% (*p* < 0.01) higher in the acute and chronic hypoxia groups, respectively, and 23.3% higher in the chronic hypoxia group than in the acute hypoxia group (*p* < 0.01). Cmax values were 13.2 and 25.6% (*p* < 0.05) lower in the acute and chronic hypoxia groups, respectively, compared to the normoxia group. When comparing the acute and chronic hypoxia groups to the normoxia group, no statistically significant change in Vd/F was observed due to inter-individual variations. However, it tended to enlarge with increasing hypoxic time and was 25.0 and 26.7% higher in the acute and chronic hypoxia groups, respectively, compared to the normoxia group. When comparing the acute and chronic hypoxia groups to the normoxia group, no statistically significant change in CL/F was observed. However, it tended to diminish with increasing hypoxic time and was 18.6% lower in the chronic hypoxia groups than in the normoxia group. When comparing the acute and chronic hypoxia groups to the normoxia group, no statistically significant change in AUC_0-12_ was observed. However, it tended to enlarge with increasing hypoxic time and was 25.6% higher in the chronic hypoxia groups than in the normoxia group. For Tmax values, we found no significant differences when comparing the acute and chronic hypoxia groups to the normoxia group.

### Protein Expression of UGT1A1 and OCT2

**FIGURE 5:**
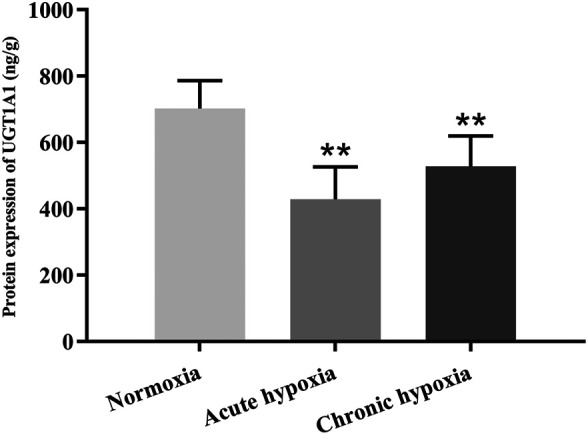
Protein expression of UGT1A1 in rats after exposure to simulated high altitude hypoxia. The data are presented as mean ± SD. *N* = 6. The data were analyzed using ANOVA, and the differences between the means of two groups were compared using LSD tests. **p < 0.01 compared to the normoxia group.

**FIGURE 6:**
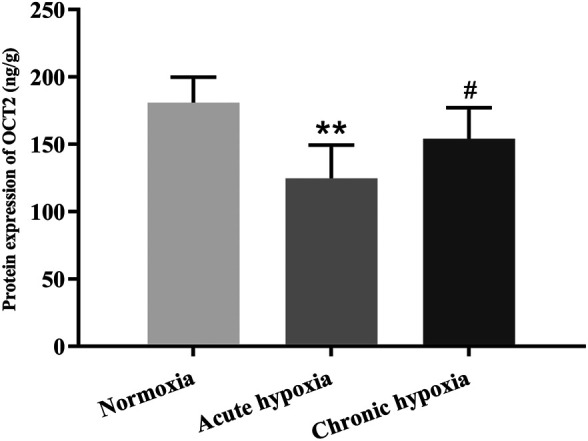
Protein expression of OCT2 in rats after exposure to simulated high altitude hypoxia. The data are presented as mean ± SD. *N* = 6. The data were analyzed using ANOVA, and the differences between the means of two groups were compared using LSD tests. **p < 0.01 compared to the normoxia group; ^#^ *p* < 0.05 compared to the acute hypoxia group.

The protein expression of UGT1A1 in the liver and OCT2 in the kidney were significantly decreased in rats after exposure to high altitude hypoxia. Figures 5, 6 show changes in the protein expression of UGT1A1 and OCT2 in rats, respectively. Relative to the normoxia group, UGT1A1 protein expression were 38.9 and 24.7% lower for the acute and chronic hypoxia groups (both *p* < 0.01), respectively. The OCT2 protein expression were 31.1% lower for the acute hypoxia group than the normoxia group (*p* < 0.01), and 23.6% higher for the chronic hypoxia group than the acute hypoxia group (*p* < 0.05).

### mRNA Expression of UGT1A1 and OCT2

**FIGURE 7:**
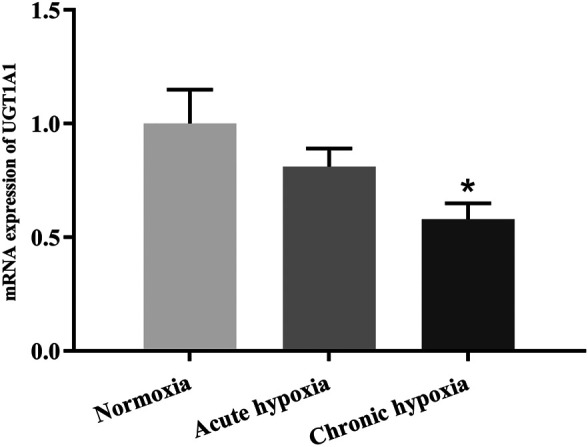
The mRNA expression of UGT1A1 in rats after exposure to simulated high altitude hypoxia. The data are presented as mean ± SD. *N* = 6. The data were analyzed using ANOVA, and the differences between the means of two groups were compared using LSD tests. *p < 0.05 compared to the normoxia group.

**FIGURE 8:**
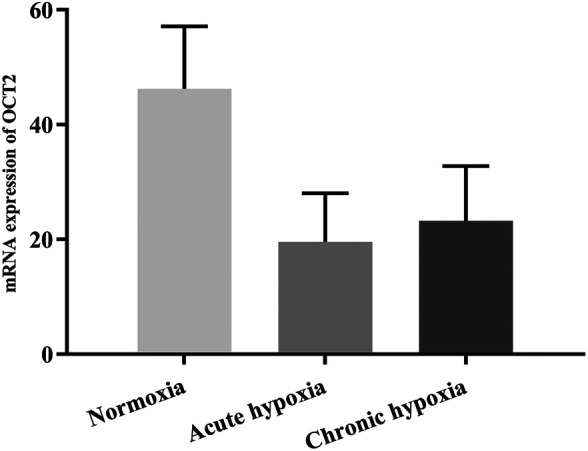
The mRNA expression of OCT2 in rats after exposure to simulated high altitude hypoxia. The data are presented as mean ± SD. *N* = 6. The data were analyzed using ANOVA, and the differences between the means of two groups were compared using LSD tests.

The mRNA expression of UGT1A1 was significantly decreased by high altitude hypoxia in rats, while the mRNA expression of OCT2 tend to be decreased despite no statistically significant changes. Figures 7, 8 show changes in the mRNA expression of UGT1A1 and OCT2 in rats after exposure to high altitude hypoxia, respectively. The mRNA expression of UGT1A1 was 42.0% lower in the chronic hypoxia group than the normoxia group (*p* < 0.05). When comparing the acute and chronic hypoxia groups to the normoxia group, no statistically significant changes in the OCT2 mRNA expression levels was observed. However, it tended to diminish and was 57.7 and 49.7% lower in the acute and chronic hypoxia groups, respectively, compared to the normoxia group.

## Discussion

In this study, the pharmacokinetic parameters of t_1/2_ and CL/F found in rats at low altitude were consistent with those reported in 1989, a pharmacokinetic study of acetaminophen (Tarloff et al., 1989). But the value for Vd/F reported in the study was about 2.0 L/kg, while the value for Vd/F in our study was about 4.68 L/kg. The pharmacokinetic parameters CL/F of metformin hydrochloride were in accordance with previous study, while the t_1/2_ observed in our study was about 50% lower (Choi et al., 2010).

We found significant differences in plasma data for several pharmacokinetic parameters of acetaminophen in the normoxia group compared with acute or chronic hypoxia rats. Compared with the normoxia group, hypoxia increased the AUC of acetaminophen and decreased the CL/F. The increase of AUC after acute or chronic exposure to hypoxia is consistent with the CL/F. When comparing to the normoxia group, the pharmacokinetic parameters of t_1/2_ and MRT in the acute and chronic hypoxia groups were significantly increased. The results suggests that hypoxia could complicate the excretion of acetaminophen. The value of Tmax in the acute and chronic hypoxia groups was in accord with that found in the normoxia group. Besides, acetaminophen-gluc, acetaminophen-sulfate, acetaminophen-cys, and NAPQI are the main metabolites causing hepatotoxicity due to an overdose in plasma and liver. How does these metabolites change under hypoxia and whether it leads to hepatotoxicity under chronic hypoxia needs future study.

Statistically significant differences were also found for some of the pharmacokinetic parameters of metformin hydrochloride in three groups. The values of t_1/2_ and MRT were significantly increased in the acute and chronic hypoxia groups compared to the normoxia group. The pharmacokinetic parameters of AUC, CL/F, and Tmax in the acute and chronic hypoxia groups were in accordance with that found in the normoxia group. When comparing the acute and chronic hypoxia groups to the normoxia group, there was no statistically significant change in the Vd/F of metformin hydrochloride due to interindividual variations. However, Vd/F tended to enlarge with the increasing of the degree of hypoxia. There are many factors affecting the pharmacokinetic parameters of acetaminophen and metformin hydrochloride under hypoxic environment, such as OCT2, UGT1A1, and physiological indexes of the body. Hypoxia may affect blood circulation (Dunham-Snary et al., 2017), vascular permeability (Marsboom and Rehman, 2018) and drug plasma protein binding rate (Arancibia et al., 2005), which may lead to the change of the apparent volume of distribution. Therefore, Vd/F of acetaminophen and metformin hydrochloride was changed in rat plasma after exposure to simulated high altitude hypoxia due to above factors. Mean plasma concentration-time profiles for metformin hydrochloride in the acute and chronic hypoxia groups seem to just shift parallelly downward, which may demonstrated that Vd/F is the only factor needed to describe the effect of hypoxia. Our results are reminiscent of the effect of hypoxia on the pharmacokinetics of ibuprofen and propranolol reported in recent studies. Acute hypoxia increased the t_1/2_ and MRT of ibuprofen in rats by 42 and 51%, respectively (Gola et al., 2013). The main pharmacokinetic AUC, t_1/2_, MRT, and Cmax of propranolol were increased by 443, 73, 47, and 353% in rats after acute exposure to high altitude, respectively, whereas Tmax and CL/F were decreased by 81 and 69%, respectively (Li W. B. et al., 2015). Our previous study indicated that the AUC of lidocaine hydrochloride and sulfamethoxazole was significantly increased, and the CL/F was significantly decreased in these healthy male subjects after either acute or chronic exposure to an altitude of ∼3,780 m in comparison to those residing at an altitude of ∼400 m (Whayne, 2014; Zhang et al., 2016).

The body’s physiologic and blood parameters are the most sensitive to hypoxia (Zhang and Li, 2015; Zhou et al., 2018). Previous studies have shown that total hemoglobin mass increased by 11% as well as a corresponding increase in red cell volume, hemoglobin concentration and hematocrit in subjects after long-term exposure to intermittent hypoxia (Katja et al., 2003). Our results show that ScO_2_ value was 7.8 and 7.8% lower, and red blood cell count were 34.9 and 14.0% higher in rats after exposure to acute and chronic hypoxia, respectively. In addition, the hemoglobin was 10.3% higher, total bilirubin was 33.3% higher, total protein was 7.6% lower, and albumin was 8.8% lower in rats after exposure to chronic hypoxia compared to normoxia rats. The rate of protein binding of acetaminophen was 20% (Debie et al., 1996), and the decreases of total protein and albumin might reduce the protein binding of acetaminophen in the condition of hypoxia. Metformin hydrochloride does not bind with plasma protein (Chaves et al., 2018), so albumin is not a factor affecting its distribution and elimination. Other studies have reported that protein binding of meperidine and furosemide decreased after exposure to high altitude hypoxia (Ritschel et al., 1996; Arancibia et al., 2004). The effect of hypoxia on the physiologic are extremely complex and not completely understood. Whether the protein binding rate of acetaminophen and metformin hydrochloride changes under hypoxia environments and influence their pharmacokinetics needs further investigation.

Acetaminophen is metabolized mainly by UGT1A1 in the liver (Wang et al., 2011). The metabolism of metformin hydrochloride are mainly affected by OCT2 (Kimura et al., 2005). This study found significant changes in the pharmacokinetics of acetaminophen and metformin hydrochloride in rats after exposure to simulated high altitude hypoxia, and they might be due to significant decreases in the expressions of UGT1A1 and OCT2. We found the protein expression levels of UGT1A1 statistically significant decreased in rats after exposure to acute and chronic hypoxia, and the changes in the protein expression levels observed echo changes in the mRNA expression levels. When comparing the acute and chronic hypoxia groups to the normoxia group, the protein expression levels of OCT2 were significantly decreased. However, there was no statistically significant change in the mRNA expression levels due to interindividual variations. Our previous studies showed that the activity, protein expression, and mRNA expression of rat CYP1A2 were significant decreased under acute and chronic high altitude hypoxia (Li et al., 2014a). In addition, the activity and protein and mRNA expression of rat CYP2E1 and CYP3A1 were significant decreased under chronic high altitude hypoxia *in vivo*. The activity, protein or mRNA expression of rat CYP2E1 or CYP3A1 did not change under acute high altitude hypoxia (Li et al., 2014b). The changes of other metabolic enzymes such as SULT1A1, SULT1A3/SULT1A4, estrogen sulfotransferase, bile salt sulfotransferase involved in the metabolism of acetaminophen under hypoxia also need to be claimed in the future.

Drug transporters also participate in the absorption, distribution, metabolism, and excretion of drugs. Several transporters are found in the small intestine, kidney, liver, and the blood-brain barrier, which play an important role in drug transport *in vivo*. Transporters in the small intestine are mainly responsible for expelling drugs and their metabolites. The transporters on the proximal convoluted tubules of the kidney can mediate partial drug excretion from urine. Transporters in liver cells are responsible for transporting drugs from blood to liver, promoting the metabolism and excretion of drugs (Song et al., 2021). The uptake transporter for acetaminophen into the hepatocyte might be downregulated by hypoxia leading to less acetaminophen entering the cell and consequently a downregulation of UGT1A1. The efflux transporter for the metabolites acetaminophen-gluc and acetaminophen-sulfate in the liver or kidney, namely MRP3, MRP4, BCRP, might be downregulated by hypoxia leading to less release of the metabolites from the liver and a possible product inhibition and downregulation of UGT1A1, or less excretion of the metabolites by the kidneys leading to a product inhibition of the efflux transporters in the liver and a product inhibition and downregulation of UGT1A1. OAT1, OAT2, and OAT3 responsible for transporting drugs from blood to proximal convoluted tubules might be downregulated then caused elevated level of metabolites of acetaminophen in the plasma. But how do they actually alter under hypoxia needs further study. Previous studies showed that the change of drug transporters under hypoxic conditions are related to the type of different tissues and exposure time to hypoxia. The expression of MDR1, MRP2, PEPT1, OATP1B1, OAT1, and OCT1 were significantly increased in the liver, and the expression of OATP2 and BCRP did not change significantly. The expression of MRP2, PEPT1, OATP1B1, OAT1, and OCT1 were increased significantly in the intestine, while the expression of MDR1 was decreased significantly. The expression of MDR1, PEPT1, OAT1, and OCT1 were increased significantly in the kidney. The expression of MRP2 was increased at first and then decreased with the hypoxia exposure time, and the expression of OATP1B1 was decreased with the hypoxia exposure time and then did not change significantly (Duan et al., 2021). Therefore, it is hard to claim how transporters alter then influence the metabolism of drugs under hypoxia due to its complex process. The effects of hypoxia on drug transporters needs further study to confirm.

Although the prevention and treatment of high altitude illness has made great progress, the clinical rational use and the efficacy of drugs has not yet caused enough attention at high altitude, so the present study is timely. We found significant changes in the disposition of acetaminophen and metformin hydrochloride in rats after exposure to simulated high altitude hypoxia, which provides a reason for considering adjustments to drug dosage in high altitude hypoxic environment. It is recommended that patients with acetaminophen or metformin hydrochloride administered at high altitudes should be closely monitored and the dose should be reduced according to changes in plasma concentration. The pharmacokinetics of acetaminophen and metformin hydrochloride at high altitude should be rechecked, and the optimal acetaminophen and metformin hydrochloride dose should be reevaluated, and adjusted if necessary. The effect of high altitude hypoxia on systemic pharmacokinetics of other drugs clearly needs further clinical evaluation.

## Data Availability

The raw data supporting the conclusions of this article will be made available by the authors, without undue reservation.
